# Supplementary material for: Single-cell microRNA-mRNA co-sequencing reveals non-genetic heterogeneity and mechanisms of microRNA regulation
Source: Nat Commun. 2019 Jan 9;10:95. doi: 10.1038/s41467-018-07981-6 (PMC6327095; doi:10.1038/s41467-018-07981-6)
Supplement: Supplementary file 1 — Supplementary Information [file 41467_2018_7981_MOESM1_ESM.pdf]

Supplementary Information

Single-cell microRNA-mRNA co-sequencing reveals non-genetic heterogeneity and mechanisms of microRNA regulation

Wang *et al.*

Supplementary Figures

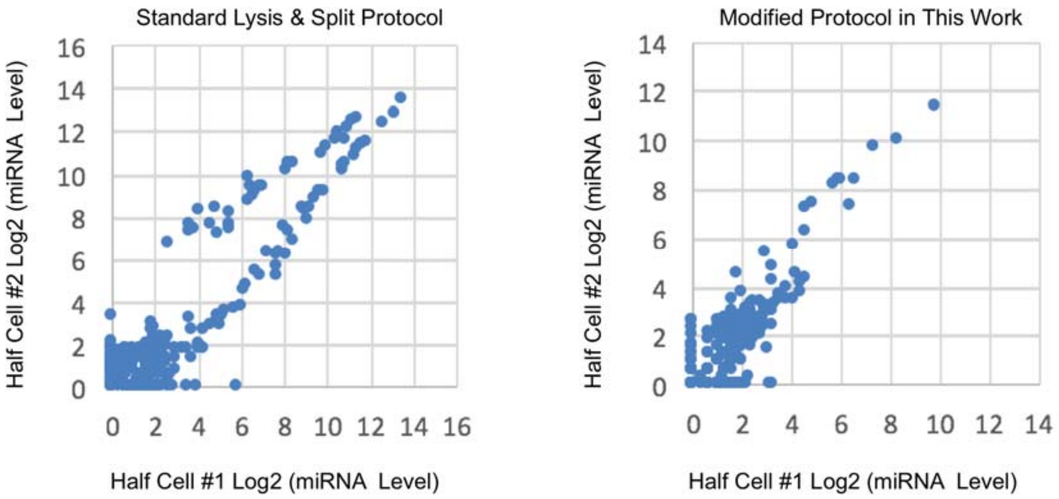

Supplementary Figure 1. New protocol to improve the splitting of single-cell lysate to profile miRNAs. Direct splitting of single-cell lysate prepared using current methods did not yield even distribution of miRNAs in two half-cell samples. This is presumably due to the binding of miRNAs in cytoplasmic proteins that are still tethered to partially digested cellular materials. The new protocol that combines multiple freeze/thaw and heat treatment led to significant improvement in single-cell lysate splitting. Left: profiling of microRNAs from two half-cell samples split from the same single cell. Cell lysis was performed using standard methods. Right: the same profiling except that the protocol for the cell lysis and pre-treatment method has been modified for single cell miRNA profiling. The log2 transformed miRNA expression levels (see Methods) are plotted, with each dot representing a single miRNA.

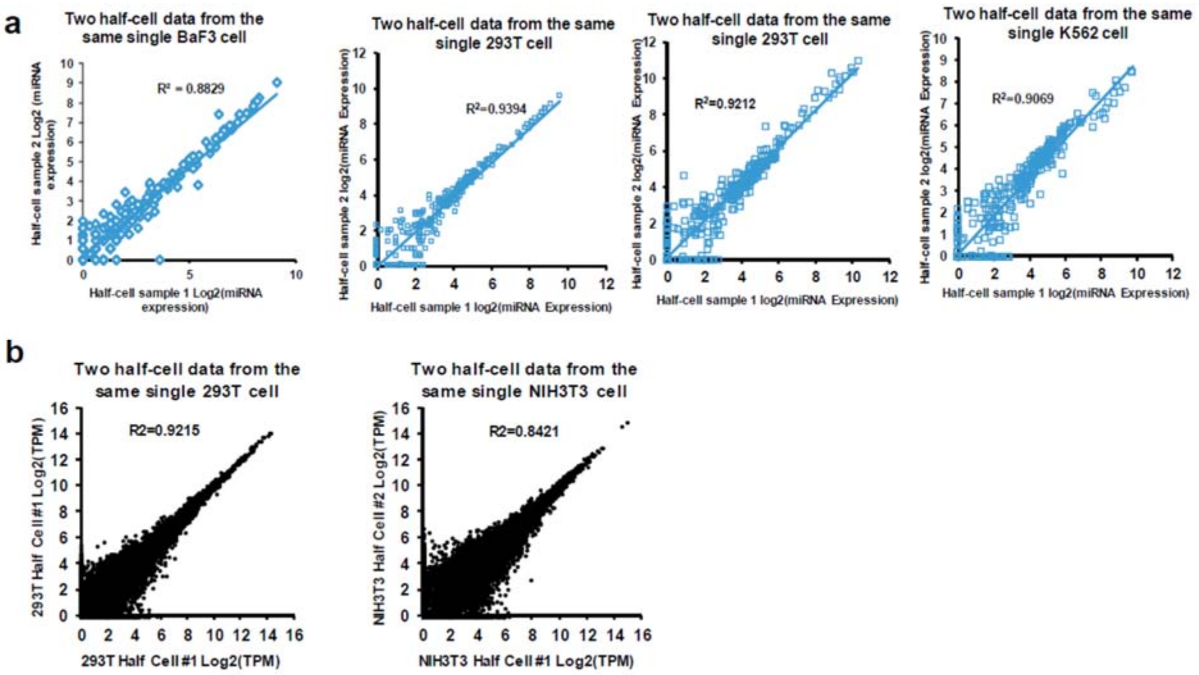

Supplementary Figure 2. Profiling of miRNA expression in two half-cell samples derived from the same single cells. (a) The indicated single cells were lysed, and two halves of the lysate from the same single cell were split (using the improved approach) and independently subjected to the processing shown in Figure 1b and then miRNAs were measured on the Luminex Multiplex Bead Array platform. Scatter plots of normalized and log2-transformed miRNA expression levels (see Methods) are shown, with each panel showing data from one of the indicated cells. Each dot represents one annotated miRNA, with R^2 indicated. **(b)** Similar to (a), the indicated single cells were lysed, and two halves of the lysate from the same single cell were split and subjected to RNAseq analyses. Scatter plots of log2-transformed mRNA expression levels are shown. Each dot represents one annotated mRNA, with R^2 indicated.

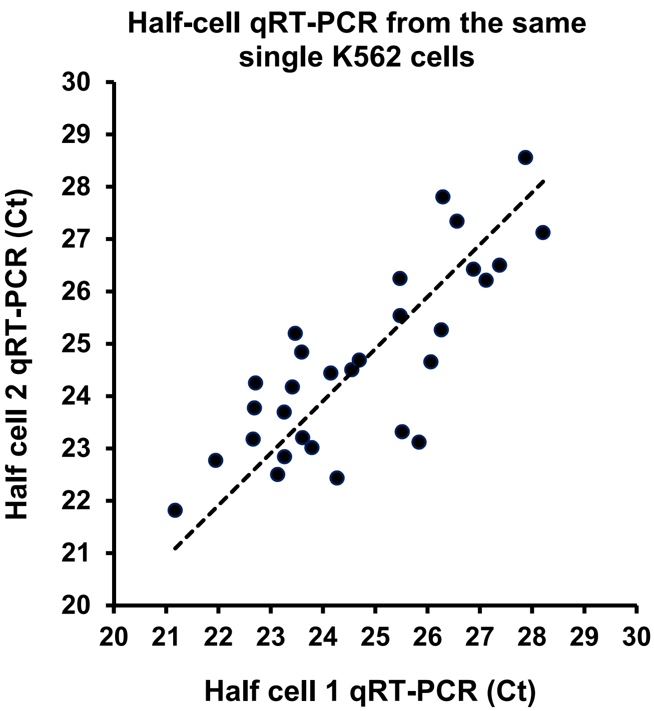

Supplementary Figure 3. qRT-PCR analysis of miRNA expression in two half-cell samples derived from the same single cells. Single K562 cells were lysed. Lysate was split with each half of the lysate analyzed for miR-146b-5p expression using qRT-PCR. Each dot reflects a single cell. A total of 30 cells were analyzed. The Scatter plot shows the Ct values (see Methods on single cell qRT-PCR) of the two halves of the same single cells.

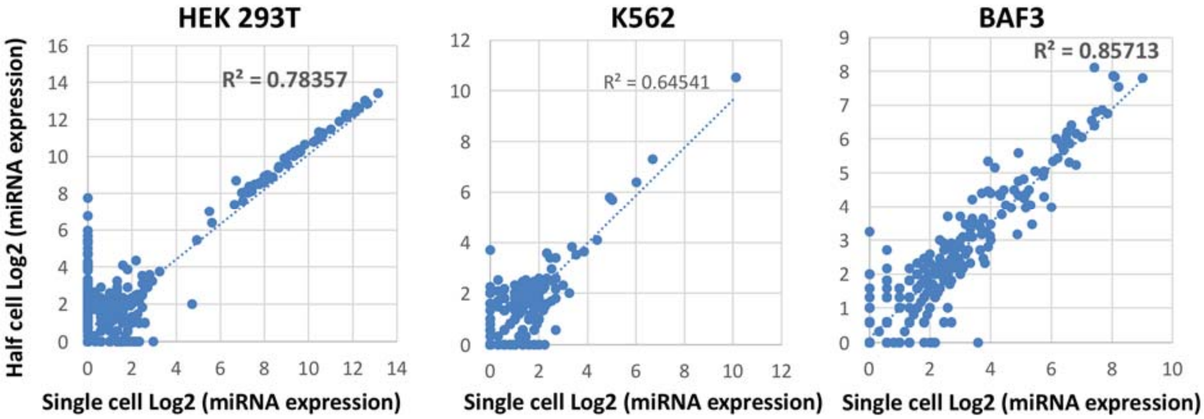

Supplementary Figure 4. Single-cell vs half-cell miRNA profiles. Scatter plots showing the comparison of miRNA profile between a single cell and a half cell cross three different types of cell lines. Cell types and R^2 values are indicated. Log2-transformed miRNA expression data are shown (see Methods), with each dot presenting a single miRNA.

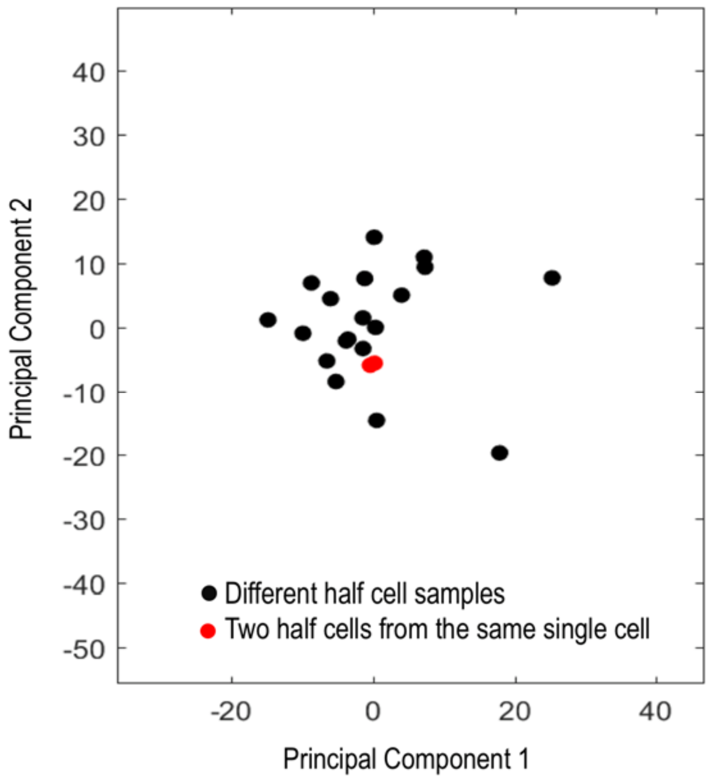

Supplementary Figure 5. Principal component analysis of half-cell miRNA profiles. miRNA expression profiles from 19 different half-cells (black) and two half-cell miRNA profiles from the same single cell (red) were analyzed with principle component analysis, with each dot representing a half cell. Note that the two red dots are very close by.

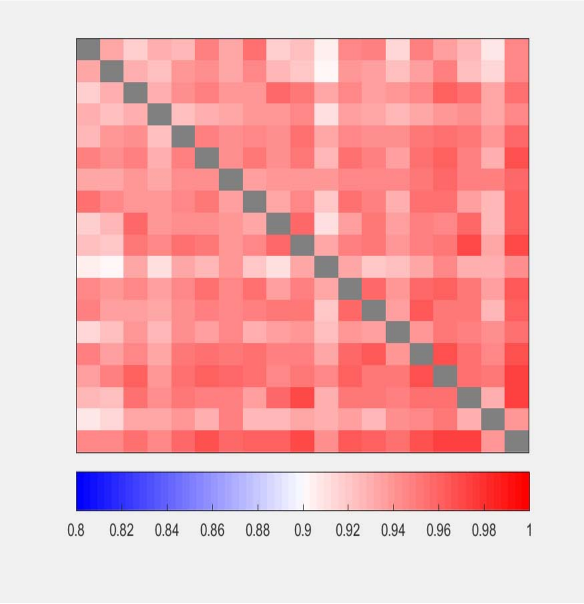

Supplementary Figure 6. Pair-wise correlation across 19 half-cell miRNA profiles. Paired half-cell mRNA and miRNA sequencing was successfully performed for 19 single K562 cells. The heatmap shows the pair-wise correlation matrix of 19 K562 half-cell microRNA data subjected to hierarchical clustering. Color key is shown below the plot as the Pearson Correlation Coefficient R. A grey color was assigned to designate self-correlations. Overall, there is a good correlation ranging from R=0.902 to 0.975 among the group of 19 single cells. The order of cells is the same as in Supplementary Figure 7.

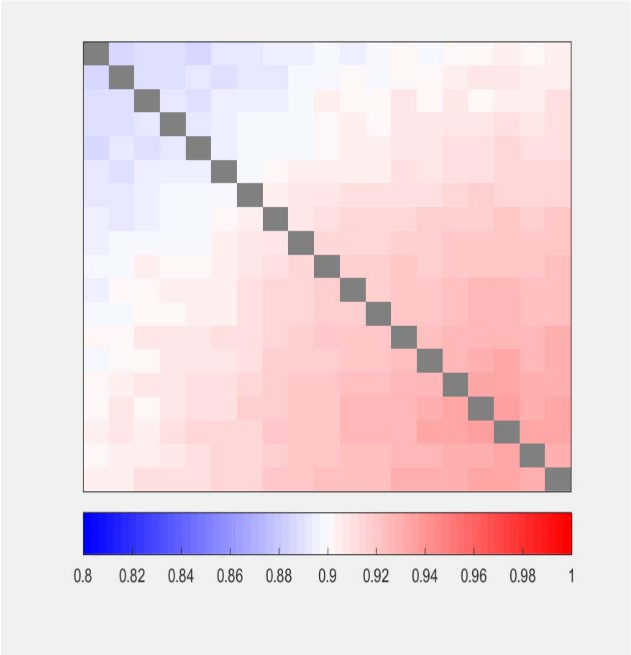

Supplementary Figure 7. Pair-wise correlation across 19 half-cell mRNA profiles. Paired half-cell mRNA and miRNA sequencing was successfully performed for 19 single K562 cells. The heatmap shows the pair-wise correlation matrix of half-cell mRNA data subjected to hierarchical clustering. Color key is shown below the plot as the Pearson Correlation Coefficient R. A grey color was assigned to designate self-correlations. The R value ranges from R=0.884 to 0.937 among the group of 19 single cells. The order of cells is the same as in Supplementary Figure 6.

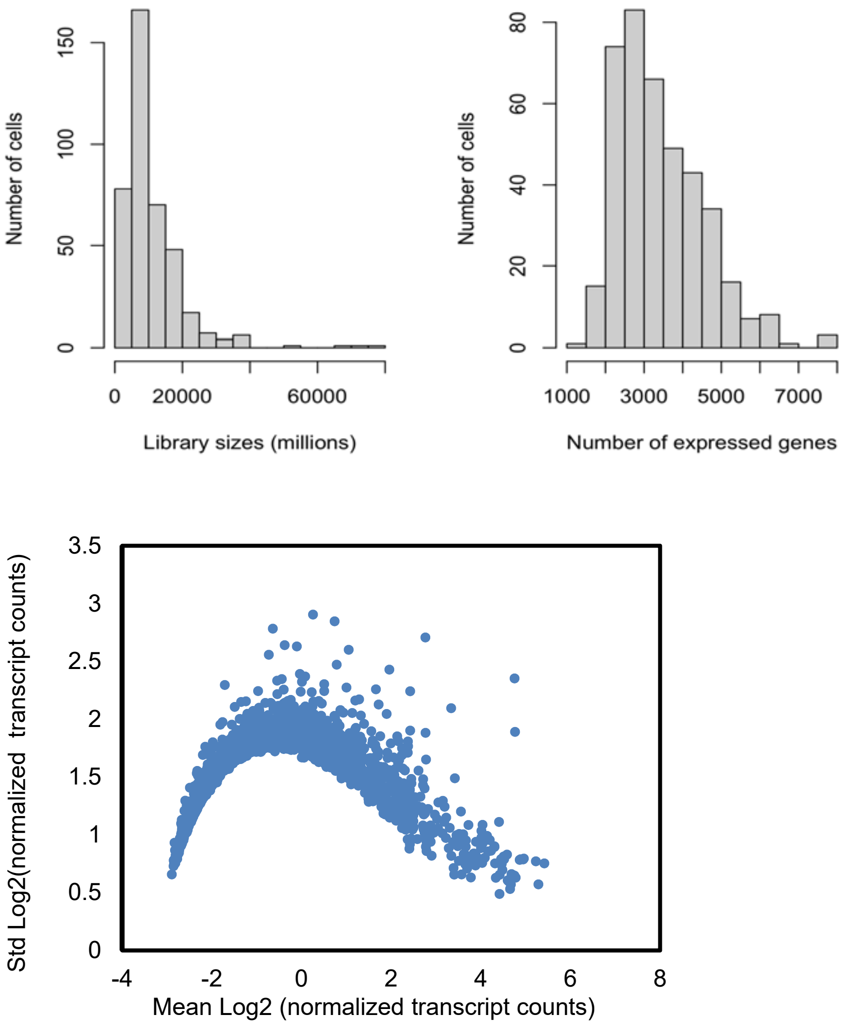

Supplementary Figure 8. Library quality check for 400 single K562 transcriptome sequencing data. Single K562 cells were analyzed using our in-house massively parallel single-cell 3'-end RNAseq technology. The quality of the libraries was examined through histograms of single cell RNAseq data as a function of the library size (reflecting total number of mapped reads, top left) or of the number of genes detected (top right). These results are in agreement with literature results[1].

The relationship between mean log2 normalized transcript counts and variation is shown

in the bottom figure with each dot representing a single detected gene. Normalization procedure is described in Methods.

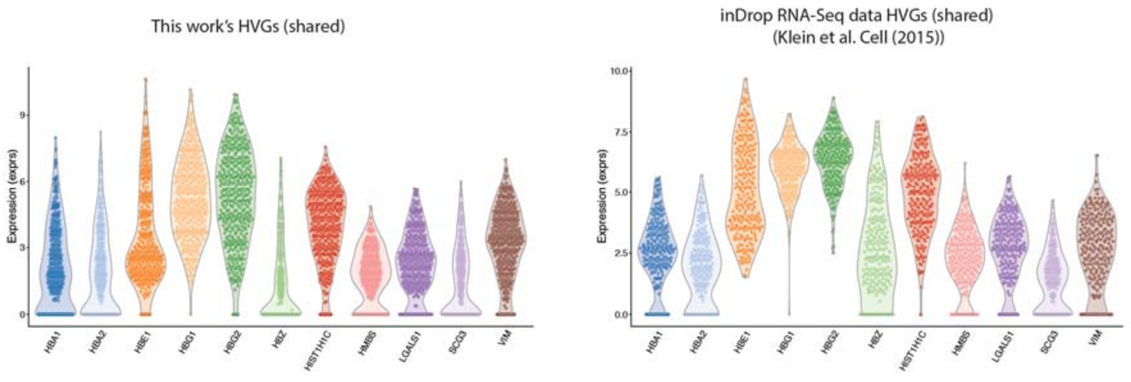

Supplementary Figure 9. Comparison of K562 single-cell mRNA-seq data between this work and the data generated by InDrop. Single K562 cells were analyzed using our in-house massively parallel single-cell 3'-end RNAseq technology (this work). The top 20 highly variable genes (HVGs) were determined from our dataset (this work, 400 single cells) and that from the data generated with InDrop published by Klein et al. (right panel, 238 single cells). The two panels show scattered and violin plots for 11 HVGs that were shared between the two datasets. Despite higher number of single cells (N=400) in this work compared to InDrop data (N=238) and the resultant lower depth per cell, the expression levels of these HVGs are consistent between this work and the InDrop K562 data.

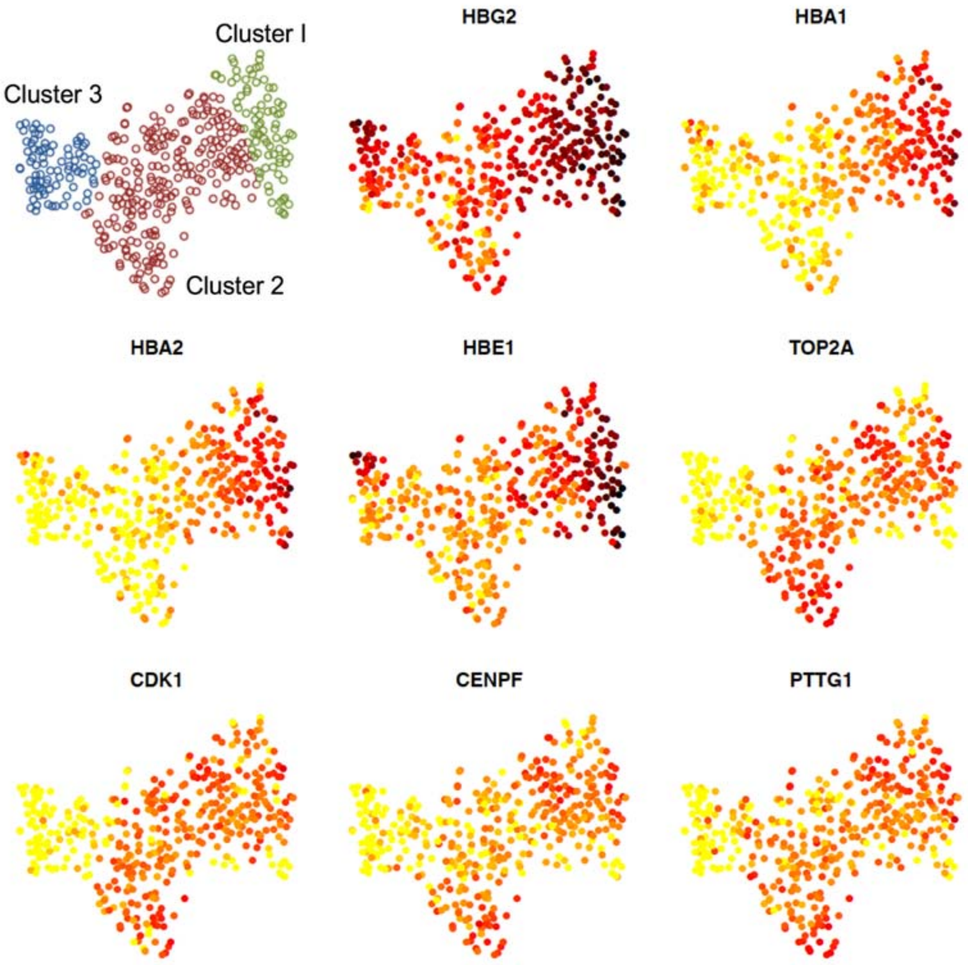

Supplementary Figure 10. tSNE Analysis of 400 single K562 cell mRNA sequencing data. Single K562 cells were analyzed using our in-house massively parallel single-cell 3'-end RNAseq technology. tSNE analysis was performed to visualize the transcriptomic data from 400 single cells. The result indicates the existence of three clusters, consistent with the heatmap in Figure 3d. Cluster memberships are indicated in the top left panel. Also shown is the distribution of selected highly variable genes in order to visualize the expression of indicated genes in major clusters. Darker colors indicate higher expression.

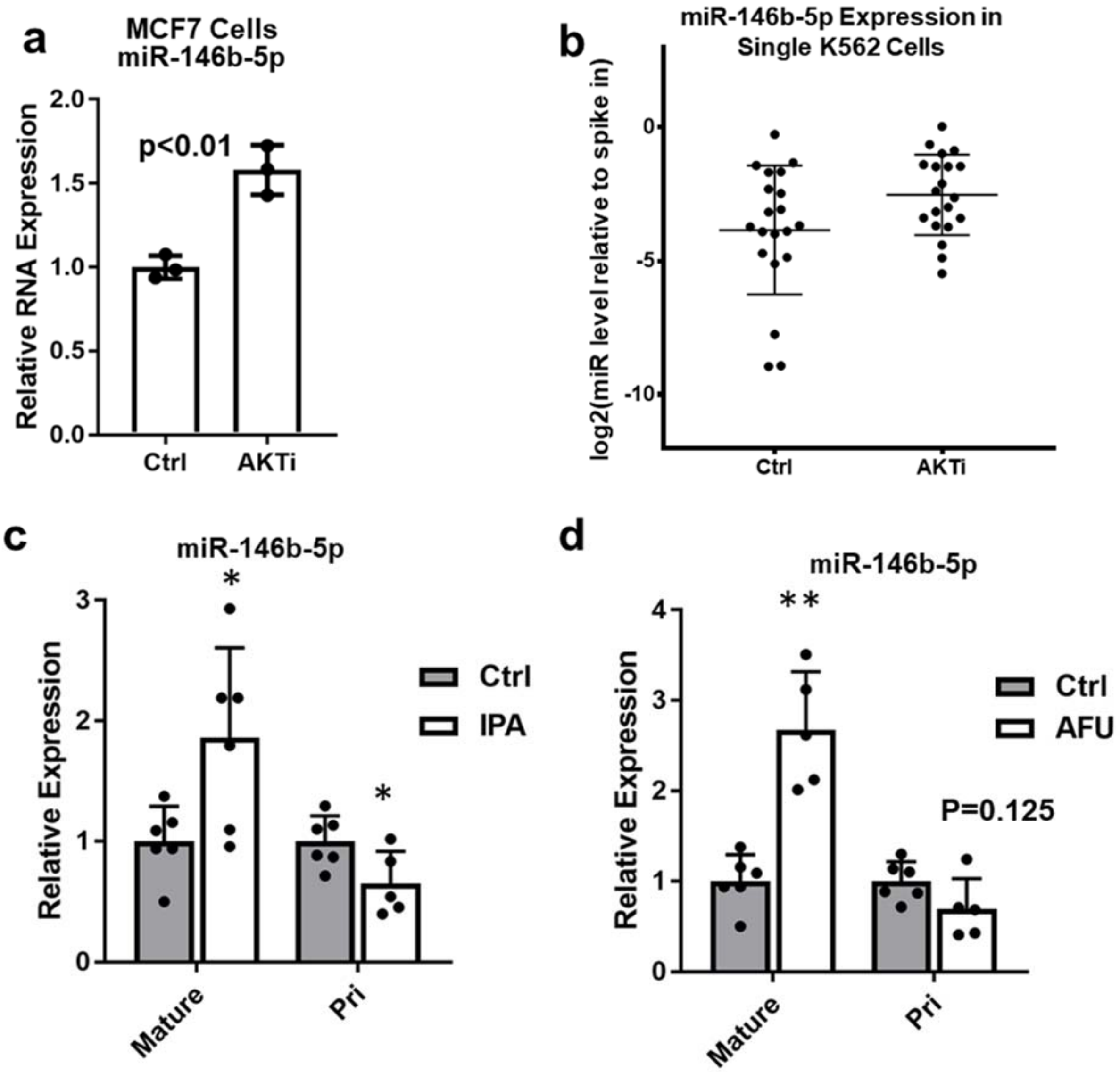

Supplementary Figure 11: The regulation of miR-146b expression by AKT. (a) MCF7 cells were treated with vehicle control (Ctrl, DMSO) or AKT inhibitor (AKTi, MK2206) for 24 hours, and the expression of miR-146b-5p was determined by qRT-PCR. N=3 biological replicates. Error bars stand for standard deviation. Data are from a representative experiment out of two performed. P<0.01, student's t-test. **(b)** K562 cells were treated with vehicle control (DMSO) or AKTi MM2206 for 24 hours. Single K562 cells were analyzed for miR-146b expression using qRT-PCR. Each dot stands for one cell.

Standard deviation in the Ctrl group is 2.41 and in the AKTi group is 1.50. Error bars stand for standard deviation. $P<0.01$ for comparing the variability of the Ctrl and the AKTi groups, using permutation tests, see Methods. **(c, d)** K562 cells were treated with two additional AKT inhibitors IPA (c) and AFU (d). The expression of mature miR-146b-5p or primary miR-146b were determined by qRT-PCR. N=5 or 6 biological replicates. Data are from a representative experiment out of two performed. Error bars stand for standard deviation. *$P<0.05$; **$P<0.01$, student's t-test.

Supplementary References

1. Klein AM et al. Droplet barcoding for single-cell transcriptomics applied to embryonic stem cells. *Cell*, 161(5), 1187-1120 (2015)
