# Supplementary material for: Single-cell microRNA-mRNA co-sequencing reveals non-genetic heterogeneity and mechanisms of microRNA regulation
Source: Nat Commun. 2019 Jan 9;10:95. doi: 10.1038/s41467-018-07981-6 (PMC6327095; doi:10.1038/s41467-018-07981-6)
Supplement: Supplementary file 3 — Description of Additional Supplementary Files [file 41467_2018_7981_MOESM3_ESM.pdf]

Description of Additional Supplementary Files

File Name: Supplementary Data 1
Description: Pathway analysis of the first & second cell clusters from the 19 K562 half-cell data. Pathway analyses were performed using DAVID comparing the gene expression between the first and second clusters of cells from the 19 K562 half-cell experiment. Enriched pathways are listed, with the table sorted by p-values (low to high).

File Name: Supplementary Data 2
Description: Pathway analysis of the first & second cell clusters from the 400 K562 single-cell data. Pathway analyses were performed using DAVID comparing the gene expression between the first and second clusters of cells from the 400 K562 single-cell experiment. Enriched pathways are listed, with the table sorted by p-values (low to high).

File Name: Supplementary Data 3
Description: Gene set enrichment analysis (GSEA) to identify pathways associated with miR-146b-5p and let-7i-5p. mRNA genes were sorted based on their correlation with miR-146b-5p or let-7i-5p in the 19 K562 half-cell data. These rank order lists were queried against MSigDB using GSEA. Top 10 results are shown.

File Name: Supplementary Data 4
Description: CMAP query with let-7i-5p mRNA signatures. mRNA genes that are correlated or anti-correlated with let-7i-5p expression were used to query the CMAP database. The results are shown.

File Name: Supplementary Data 5
Description: Summary of small RNAs in 19 K562 half-cell data that mappable to other genomic regions. Read counts of small RNAs that were mapped to other RNA types, from the 19 K562 half-cell data.

File Name: Supplementary Data 6
Description: List of oligonucleotides and primer sequences, with their respective modifications. Modifications are annotated using IDT nomenclature.

File Name: Supplementary Software 1
Description: Matlab codes are provided that analyze the co-expression between miRNA and mRNAs profiles from the 19 K562 cells. A README file is also included with instructions.
