# Supplementary material for: Single-cell microRNA-mRNA co-sequencing reveals non-genetic heterogeneity and mechanisms of microRNA regulation
Source: Nat Commun. 2019 Jan 9;10:95. doi: 10.1038/s41467-018-07981-6 (PMC6327095; doi:10.1038/s41467-018-07981-6)
Supplement: Supplementary file 11 — Reporting Summary [file 41467_2018_7981_MOESM11_ESM.pdf]

nature research

Corresponding author(s): Rong Fan
Jun Lu

Reporting Summary

Nature Research wishes to improve the reproducibility of the work that we publish. This form provides structure for consistency and transparency in reporting. For further information on Nature Research policies, see Authors & Referees and the Editorial Policy Checklist.

Statistical parameters

When statistical analyses are reported, confirm that the following items are present in the relevant location (e.g. figure legend, table legend, main text, or Methods section).

n/a	Confirmed	
☐	☒	The exact sample size (*n*) for each experimental group/condition, given as a discrete number and unit of measurement
☐	☒	An indication of whether measurements were taken from distinct samples or whether the same sample was measured repeatedly
☐	☒	The statistical test(s) used AND whether they are one- or two-sided<br>*Only common tests should be described solely by name; describe more complex techniques in the Methods section.*
☐	☒	A description of all covariates tested
☐	☒	A description of any assumptions or corrections, such as tests of normality and adjustment for multiple comparisons
☐	☒	A full description of the statistics including central tendency (e.g. means) or other basic estimates (e.g. regression coefficient) AND variation (e.g. standard deviation) or associated estimates of uncertainty (e.g. confidence intervals)
☐	☒	For null hypothesis testing, the test statistic (e.g. *F*, *t*, *r*) with confidence intervals, effect sizes, degrees of freedom and *P* value noted<br>*Give P values as exact values whenever suitable.*
☒	☐	For Bayesian analysis, information on the choice of priors and Markov chain Monte Carlo settings
☒	☐	For hierarchical and complex designs, identification of the appropriate level for tests and full reporting of outcomes
☒	☐	Estimates of effect sizes (e.g. Cohen's *d*, Pearson's *r*), indicating how they were calculated
☐	☒	Clearly defined error bars<br>*State explicitly what error bars represent (e.g. SD, SE, CI)*

Our web collection on statistics for biologists may be useful.

Software and code

Policy information about availability of computer code

Data collection	Luminex xPonent (v3) software was used to acquire data on the Luminex instrument. Real time PCR data were acquired using CFX Manager software (v2) on Biorad CFX machines. Illumina software (CASAVA-1.8.2) was used to acquire data on Illumina sequencing machine.
Data analysis	GenePattern, v3, was used to analyze RNAseq data; miRDeep2-based perl codes to analyze small RNA sequencing data (these codes have been described in our previous publication); GSEA v2.0, for performing gene set enrichment analysis; DAVID (v6.8), for performing Gene Ontology analyses; custom matlab codes were used to analyze co-expression of miRNA and mRNA (provided as a zip file). All stated in methods.

For manuscripts utilizing custom algorithms or software that are central to the research but not yet described in published literature, software must be made available to editors/reviewers upon request. We strongly encourage code deposition in a community repository (e.g. GitHub). See the Nature Research guidelines for submitting code & software for further information.

Data

Policy information about availability of data

All manuscripts must include a data availability statement. This statement should provide the following information, where applicable:

- Accession codes, unique identifiers, or web links for publicly available datasets
- A list of figures that have associated raw data
- A description of any restrictions on data availability

Next-generation sequencing data have been deposited to GEO under series GSE114071.

Field-specific reporting

Please select the best fit for your research. If you are not sure, read the appropriate sections before making your selection.

☒ Life sciences ☐ Behavioural & social sciences ☐ Ecological, evolutionary & environmental sciences

For a reference copy of the document with all sections, see nature.com/authors/policies/ReportingSummary-flat.pdf

Life sciences study design

All studies must disclose on these points even when the disclosure is negative.

Sample size	For experiments in which sample size is relevant, the number of replicates were determined after an initial experiment with three biological replicates to gauge the data variation.
Data exclusions	One of the single cells of K562 was excluded because it failed both mRNA and miRNA sequencing. This is stated in the main text of the manuscript.
Replication	Experiments for chemical treatment of cells were performed at least twice, with similar findings. Only data from a single experiments are shown. All single cell experiments were performed with the number of cells stated.
Randomization	Experiments were designed and carried out at one performance site. Experiments were designed with relevant controls. Co-variants are not relevant.
Blinding	Binding was used during the analysis of chemical treatment effects, in which one person performed the treatment and RNA collection and another person performed qRT-PCR analysis.

Reporting for specific materials, systems and methods

Materials & experimental systems

n/a	Involved in the study
☒	☐ Unique biological materials
☒	☐ Antibodies
☐	☒ Eukaryotic cell lines
☒	☐ Palaeontology
☒	☐ Animals and other organisms
☒	☐ Human research participants

Methods

n/a	Involved in the study
☒	☐ ChIP-seq
☒	☐ Flow cytometry
☒	☐ MRI-based neuroimaging

Eukaryotic cell lines

Policy information about cell lines

Cell line source(s)	Cell lines were from American Type Culture Collection (ATCC).
Authentication	K562 cells were tested and showed sensitivity to Imatinib. BaF3 cells were tested and showed sensitivity toward IL3 withdrawal. MCF-7 cells were tested and showed sensitivity toward estrogen receptor inhibition.
Mycoplasma contamination	Cells were acquired mycoplasma-free, but have not been specifically tested for mycoplasma in this study.

Commonly misidentified lines
(See ICLAC register)

None
